# Where it all begins: Predicting initial therapeutic skills before clinical training in cognitive behavior therapy

**DOI:** 10.1371/journal.pone.0294183

**Published:** 2024-02-22

**Authors:** Jana Schaffrath, Jana Bommer, Brian Schwartz, Wolfgang Lutz, Ann-Kathrin Deisenhofer

**Affiliations:** Department of Clinical Psychology and Psychotherapy, University of Trier, Trier, Germany; Medical University of Vienna, AUSTRIA

## Abstract

To train novice students adequately, it is crucial to understand where they start and how they develop their skills. This study examined the impact of novice students’ characteristics on their initial clinical micro-skills when treating simulated patients with cognitive behavior therapy. The sample consisted of 44 graduate psychology students treating seven simulated patients. Clinical micro-skills were measured both using video-based ratings in reaction to short video clips of simulated patients (via the Facilitative Interpersonal Skills (FIS) performance task) and by using video-based ratings within a session with a simulated patient (using the Inventory of Therapeutic Interventions and Skills; ITIS). Two separate LASSO regressions were performed using machine learning to select potential predictors for both skills assessments. Subsequently, a bootstrapping algorithm with 10,000 iterations was used to examine the variability of regression coefficients. Using LASSO regression, we identified two predictors for clinical micro-skills in standardized scenarios: extraversion (*b* = 0.10) and resilience (*b* = 0.09), both were not significantly associated with clinical micro-skills. Together, they explained 15% of the skill variation. Bootstrapping confirmed the stability of these predictors. For clinical micro-skills in sessions, only competitiveness was excluded by LASSO regression, and all predictors showed significant instability. The results provide initial evidence that trainees’ resilience and extraversion should be promoted in the clinical training of cognitive behavior therapy. More studies on clinical micro-skills and training with larger sample sizes are needed to fully understand clinical development.

## Introduction

Over the last decades, there has been much research on the differences between therapists and the effectiveness of therapist skills training. Still, it remains unclear where novice students begin, which initial skills they already show, and how these differ inter-individually.

Investigating inter-individual differences between therapists and their relation to therapy outcome has a long tradition. In 1974, Ricks discovered that some therapists had patients with systematically better outcomes than their colleagues [1]. Consequently, the term *therapist effect* was coined to describe the therapist’s contribution to patient outcomes. Following this realization, it became a popular endeavor to find out which characteristics make more effective therapists in order to adjust training to make therapy even more effective for patients. However, not all differences between therapists are clinically relevant and associated with treatment outcome [2]. While demographic variables do not appear to influence treatment outcomes [e.g. 3, 4], the role of therapeutic experience remains vague and raises questions about the underlying learning processes [e.g. 5–7]. Things become even more complex when other therapist characteristics are examined because there is considerable heterogeneity in the studies with partly conflicting results [e.g. 8–10]; for a comprehensive review of characteristics of effective therapists see [11]. One possible explanation for these inconsistent and partially non-significant results could be that the associations between therapeutic characteristics and treatment outcome are non-linear and thus cannot be detected with methods like linear regression or correlation analysis. For example, Delgadillo and colleagues found a non-linear relation between above-average expression of agreeableness and outcome. This could indicate that it makes a difference in therapist skills when some characteristics are below average versus average, but that there are no further benefits to an above-average level of the characteristic (i.e., logarithmic relation), which should be considered in future studies [5].

The aforementioned personality traits have mostly been examined in therapists who have already completed their training or are currently enrolled in a training program. Therefore, they have already had the opportunity to practice their skills to different extents and thereby increase their effectiveness. As studies on therapist training have shown, specific training programs can increase trainees’ empathy with their clients [12], their cultural competence [13], interpersonal skills [14] as well as their self-efficacy and use of helping skills [15]. However, studies on therapist training often focus on comparing different training methods, and only a few studies describing training methods report which learning principle their training is based on [16]. Thus, it remains unknown how novice students learn therapeutic skills, whether their (professional) personality changes during training and how (and if) characteristics can distinguish between future effective and less effective therapists. The answers to these questions could help to understand the underlying processes and hence help to adapt these training methods to novice students’ needs in order to train them adequately and thereby train more effective therapists [8, 17].

One model that attempts to capture the different stages of therapeutic growth is the developing practitioner model [18]. It differentiates therapists’ professional development into five different phases: the novice student phase, the advanced student phase, the novice professional phase, the experienced professional phase, and the senior professional phase. Using this model, therapists can be studied in the different stages of their training and followed on their career paths. We believe it is important to focus on the first stage and examine which skills novice students bring along before starting their clinical training because of two main reasons. Firstly, if we want to train someone we need to know their actual level of knowledge/skills in order to neither under nor overwhelm the person with the training content. Secondly, understanding these initial or “natural” skills and how they are related to students’ other personality traits may facilitate the more targeted acquisition of competence in the long term.

According to the developing practitioner model, novice students understand theories, demonstrate initial competencies in action, and cope with their own intense emotions in the initial contact with patients. Thus, their learning process is particularly characterized by model learning, processing negative feedback, and a strong orientation toward their supervisors. Due to the intense emotions in their first patient contact, novices are preoccupied with themselves and develop a predominantly internal focus. At this stage, we would expect varying levels of skills between novice students that could be due to different coping mechanisms (e.g., processing negative feedback), differences in personal characteristics (e.g., self-confidence) or different levels of previous experience. Understanding these initial differences would provide the opportunity for adapting training to trainees’ individual needs, which in turn could result in better transfer of skills.

Over the last decades, various definitions of relevant therapeutic skills or competencies have emerged to explain differences in therapists’ effectiveness. In their transtheoretical framework model, Lutz et al. [19] attempt to summarize these definitions and describe three different levels of competence.

At a macro-level, change mechanisms and principles can be described, such as Grawe’s common factors [20] or the principles of change proposed by Eubanks and Goldfried [21] that can be initiated by therapists by varying interventions.

At a meso level, specific techniques can be observed that might be typical of certain therapy orientations, e.g., exposure in CBT for anxiety disorders or chair exercises in schema therapy.

On the lowest level, they allocate basic clinical skills, or *clinical micro-skills*, such as clarity of communication, time management, paraphrasing patient statements, warmth, empathy, or triggering hope. These clinical micro-skills are demonstrated in each session across all therapy orientations. Strikingly, if therapists are asked to assess their own clinical micro-skills, no association with their patients’ outcome can be found [22]. But if these skills are assessed in sessions by trained raters (using validated rating instruments as e.g., the Cognitive Therapy Scale (CTS; Young & Beck [unpublished]) or the Inventory of Therapeutic Interventions and Skills (ITIS) [23], recent reviews and meta-analyses suggest a small but significant positive association between clinical micro-skills and therapy outcome [24, 25]. Further, these skills can also be assessed in standardized situations (e.g., with the Facilitative Interpersonal Skills (FIS) performance task [22]) and have shown positive associations with therapy outcomes [22, 26, 27]. Thus, the clinical micro-skills are well suited to study the skill acquisition of novice students, as they form the basis of all other competencies, including global principles of change or common factors that mediate positive psychotherapy outcomes. For example, proficiency in micro-skills like warmth, empathy, and instilling hope may correlate with an increased ability to establish a robust working alliance, leading to improved results.

As novice students usually do not have regular and independent patient contact, a standardized setting is required to both practice safely and examine their skills. This can be achieved using simulated patients. Simulated (or standardized) patients are “actors who stand in for and portray actual patients” [28]. The range of possible uses for simulated patients extends from short video clips of difficult patient-client situations to which therapists must respond (e.g., FIS performance task (Anderson et al. [unpublished]), to actors playing the patient for an entire treatment. Compared to real patients, this teaching method offers several advantages, including economic efficiency, the possibility to standardize learning experiences, and the elimination of risk of harm to patients as a result of unpleasant interactions with inexperienced students. In many healthcare professions, the use of simulated patients has proven its worth in the context of education and training (e.g., human medicine [29]). However, therapist training seems to be an exception and this method has become a focus of research only in the last two decades. In a study with 156 students, working with simulated patients had a positive impact on their self-efficacy, and sessions were rated as comparable to real-life interactions with patients [30]. Further studies reported a high authenticity of simulated patients in therapist training [31] and its benefits in the field of child and youth psychotherapy [32]. A study comparing competence levels between graduate students working with simulated patients and therapists in postgraduate clinical training working with “real” patients found statistically significant differences according to their different levels of professional development [33].

To date, research on therapist characteristics, therapist skills, and their training shows a lack of focus on initial clinical micro-skills and traits novice students already bring along before starting their clinical training. Understanding these initial clinical micro-skills and pre-existing traits among novice students is essential as it can provide valuable insights into tailoring more effective and personalized training programs, ultimately enhancing the overall quality of therapeutic care. Thus, the present study aims to investigate the initial clinical micro-skills–both in reactions to standardized video clips of simulated patients and during simulated therapy sessions—with which novice students embark on their careers as mental health providers and how these skills relate to their individual characteristics. The focus here is on basic competencies that can be observed in every therapy session and at every stage of professional development, and on personal characteristics previous research has found to be significantly related to better therapy outcomes (i.e., resilience, mindfulness, interpersonal problems, personality traits, empathy, self-confidence, and well-being [11]).

Clinical micro-skills ratings are based on videotaped interactions with simulated patients. As previous studies have mainly examined linear relations, in this study, the particular shape (e.g., logarithmic) of the associations of the different characteristics with clinical micro-skills is investigated.

The study was guided by the following research question: What characteristics are associated with novice students’ clinical micro-skills in reaction to short video clips of simulated patients (*Research Question 1*) and their clinical micro-skills in treating simulated patients during a therapy session (*Research Question 2*)?

## Method

### Data collection

The data was collected between April 2021 and February 2022 in seven university case seminars in a master’s program in psychology. The seminars were supervised by three licensed CBT therapists (66% female) and two female therapists in post-graduate clinical training in CBT. Three of them taught only one case seminar during this period, the other two taught one seminar in each of the two semesters. Their average experience teaching clinical classes was 8.80 semesters (*SD* = 3.56, *Range* **=** 5–13). Approximately 10 students in every seminar treated the same simulated patient supervised by the lecturer. Before the seminar, students answered questionnaires about potential predictors and reacted to short video clips of simulated patients. Additionally, during the seminar, five consecutive therapy sessions were held, and each student acted as a therapist once with the same simulated patient within each seminar. The five sessions were similar to the procedure at the beginning of outpatient psychotherapy in the German healthcare system, comprising an initial interview, a diagnostic interview, psychoeducation, planning of treatment goals, and first disorder-specific interventions. Between sessions, the progress was discussed in class and the next session was planned. They were asked for their informed consent to use the videos for research and were informed that neither their participation in the study nor their ratings would affect their grade. Data and videos were analyzed anonymously and separately so that individual participants could not be identified. Ethical approval was not obtained because the study analyses data generated during a regular university seminar.

### Participants

#### Novice students

**Fig 1:**
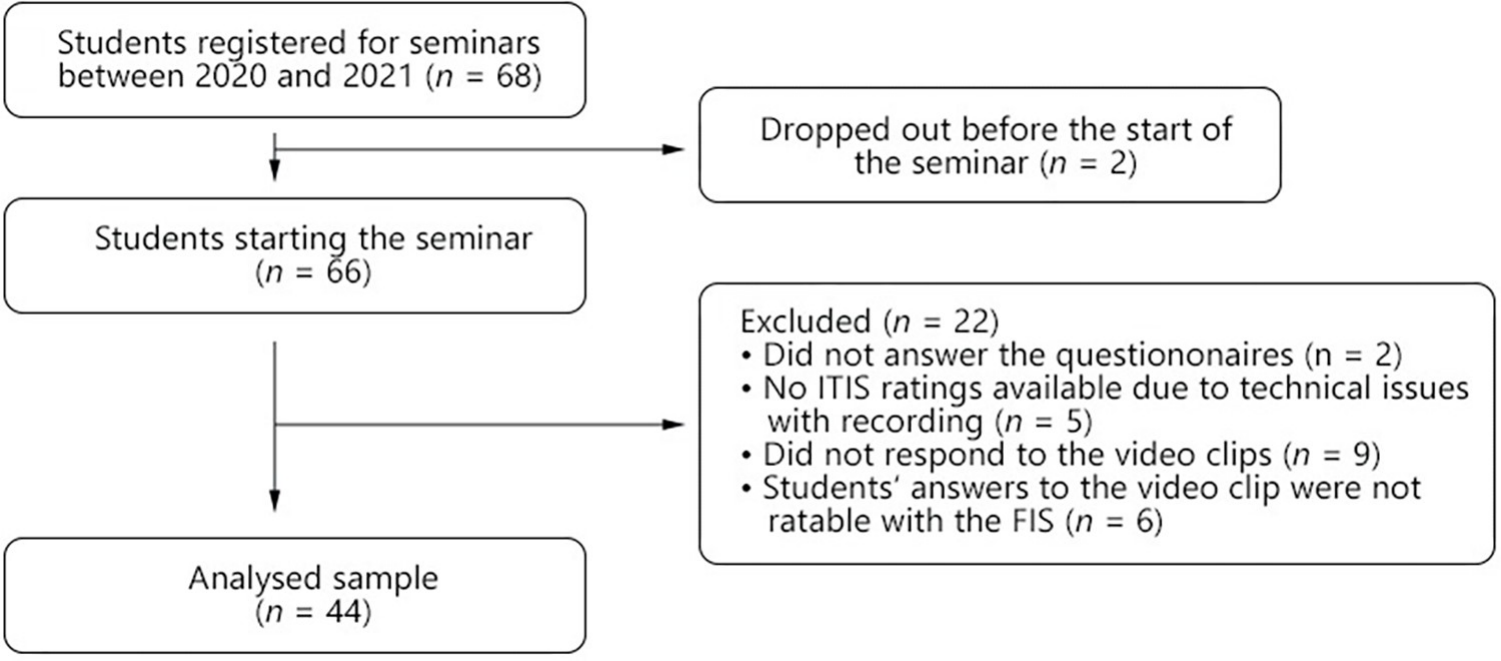
Flow chart of in- and excluded students. ITIS = Inventory of Therapeutic Interventions and Skills: used to measure clinical micro-skills in-session; FIS = Facilitative Interpersonal Skills: used to measure clinical micro-skills in standardized situations. Six students’ responses to the video clips were not ratable with the FIS because their comments were based on hypothetical considerations rather than being a direct response in the form of interaction.

Sixty-eight students were registered in the seminars between 2021 and 2022. Two students dropped out right at the beginning, so 66 students participated and acted as a therapist once during their seminar. Inclusion criteria for the study were that students gave their informed consent for the use of their data and videos for research, that they filled in the questionnaires, reacted to the video clips, and that their interaction with the simulated patient was rated with the ITIS, which reduced the sample to *N* = 44 students (Fig 1).

On average, participants were in their third semester of the master’s program in psychology (*M* = 3.41, *SD* = .62, Range = 2–5). Eighty-six percent of novice students were female (*N* = 38). Their mean age was 25.5 years (*SD* = 3.05, *Range* = 22–37). Sixty-one percent of novice students stated that they already had previous professional experience (e.g., independent interactions with patients during a clinical internship in a psychiatric clinic). The students were taught in seven different seminars. Three to nine students from each seminar were included in the study.

#### Simulated patients

During the seminars, five therapists in post-graduate clinical training (4 females) acted as simulated patients, two of whom participated in two seminars with different patient roles. They were instructed to play patients they had treated themselves but to change any details that would allow identification of the patient. The simulated patients were to be as similar to real patients treated at the outpatient clinic as possible to ensure authenticity. However, the actors were asked to leave out any contradicting information to prevent the case from becoming too complex. Thus, within seminars, the novice students all interacted with the same patient but in consecutive sessions.

### Externally assessed clinical micro-skills

#### Clinical micro-skills in standardized situations

In the first session of the seminar, students were asked to complete an adapted version of the FIS performance task [20]. The task involves participants responding to two short video clips (i.e., approximately one minute each) depicting challenging moments in therapy (i.e., patient complaining about the therapist). Each video includes a female student reenacting transcripts from simulated therapy sessions. She was coached by the research team on how to capture the style of the patients she was enacting. She was filmed by a camera that was directly facing her.

Students completed the assessment individually at home. After viewing each clip on an online platform, they were instructed to respond as if they were the therapist for the patient and video record their responses via zoom. They were not given a response length. The response length of the students varied between 30 seconds and 3 minutes. Before the assessment, students watched an explanation video of the task and had the opportunity to ask questions.

The novice students’ (*N* = 44) video recordings (*N* = 88) were rated using the German FIS Manual, which describes nine different FIS domains: a) verbal fluency; b) hope and positive expectations; c) persuasiveness; d) emotional expression; e) warmth, acceptance, and understanding; f) empathy; g) alliance bond capacity; h) alliance rupture-repair responsiveness; and i) authenticity and sincere cooperation [34]. Two trained female raters coded each domain on a 5-point Likert scale ranging from 1 (deficit) to 5 (optimal performance). The items offered operational definitions for subjective judgments about interpersonal qualities and raters underwent training on how to use the manual through calibrated examples of the levels of the scale. About every four weeks, raters met for a calibration meeting, during which divergent codes and selected examples were discussed. For this paper, the mean of both raters was taken as the estimate of the students’ scores. For the analyses, a FIS total score was calculated to estimate the novice students’ clinical micro-skills in standardized situations. After excluding the domain “authenticity and sincere cooperation” due to low reliability, the intraclass correlation was *ICC(*3, k*)* = .58 (Interpretation of mean ICC were according to Cicchetti (1994): < .40 = poor, .40 - .59 = moderate, .60 - .74 = good, .75–1.00 = excellent.).

#### Clinical micro-skills in simulated sessions

The recorded simulated therapy sessions were rated with the Inventory of Therapeutic Interventions and Skills (ITIS), which was developed to assess treatment integrity in CBT [22]. It comprises two scales, namely *Interventions* and *Skills*, rating both the correct application of techniques/strategies (e.g., the frequency and quality with which techniques and strategies are delivered, including classic and third-wave CBT interventions as well as common strategies) as well as clinical micro-skills (e.g., empathic understanding, therapeutic relationship/ collaboration). For this study, only the clinical micro-skills scale was used, which includes eleven items rated on a 7-point Likert scale from 0 (poor) to 6 (excellent). Further, overall clinical micro-skills are rated on a 7-point Likert scale ranging from 0 (poor) to 6 (excellent). Three comprehensively trained female raters evaluated the student therapists (*N* = 44) in the 23 videotaped sessions using the ITIS after the seminars were completed. For this study, two of the raters were trained in both skill rating systems. The two halves of each session were conducted by two different student therapists and therefore evaluated separately. Inter-rater reliability for clinical micro-skills ratings with the ITIS has been reported to be good (mean Kendall’s *W* = .70 [22]).

### Potential predictors of clinical micro-skills

At the beginning of the semester, the students answered questionnaires on several characteristics based on research on therapist effects [4, 5, 8, 11, 35]. The potential predictors of clinical micro-skills included: resilience, mindfulness, interpersonal problems, personality traits, empathy, self-confidence, and well-being.

Resilience was measured with the 25-item Connor-Davidson Resilience Scale (CD-RISC) [36]. Students answered 15 items on trait mindfulness using the Mindful Attention Awareness Scale (MAAS) [37]. In addition, interpersonal problems were assessed with a 12-item short version of the German version of the Inventory of Interpersonal Problems (IIP-12) [38]. The inventory captures the following four subscales: introversion, compliance, competitiveness, and self-insecurity. Personality traits were measured with the German version of the Ten Item Personality Inventory (TIPI-G) [39], which includes the five scales agreeableness, conscientiousness, openness to experience, extraversion, and emotional stability. Empathy was measured with three of four subscales of the German version of the Interpersonal Reactivity Index (IRI) [40], namely perspective taking, empathic concern, and personal distress, resulting in 21 items (seven for each subscale) [41]. Self-confidence regarding various aspects of treatment (e.g., recognizing and resolving alliance ruptures) was assessed using a scale consisting of nine items. The items were drawn from the Improving Access to Psychotherapy program and were adapted for use in this study [42]. Well-being was assessed with a one-item question (How would you most likely rate your current well-being?) on a 6-point scale ranging from ‘Very good. My life fulfills almost all my desires.’ to ‘Pretty bad. I can hardly get by.’

Descriptive statistics for all potential predictors can be found in Table 1.

**Table 1:** Descriptive statistics of all potential predictors of clinical micro-skills.

		*M*	*SD*	Range
CD-RISC	Resilience	3.63	0.49	2.4–4.6
MAAS	Mindfulness	4.28	0.70	3.1–5.6
IIP	Self-insecurity	2.85	0.86	1–5
	Competitiveness	1.90	0.66	1–3.67
	Compliance	2.50	0.74	1–4
	Introversion	2.40	0.66	1–4
TIPI-G	Openness	5.36	1.12	2–7
	Neuroticism	3.24	1.30	1–6.5
	Conscientiousness	5.85	0.84	3–7
	Agreeableness	5.64	0.87	3–7
	Extraversion	4.70	1.24	1–7
IRI	Empathy	3.89	0.43	2.9–4.6
	Well-Being	2.50	0.79	1–5
	Self-confidence	2.44	0.70	1.1–4.4

CD-RISC = Connor-Davidson Resilience Scale (36); MAAS = Mindful Attention Awareness Scale (37); IIP = Inventory of Interpersonal Problems (38); TIPI-G = Ten Item Personality Inventory—German version (39); IRI = Interpersonal Reactivity Index (40). *N* = 44 students.

### Analyses

The statistical analyses were conducted using RStudio version 2022.07.1+554 [43] and R version 4.1.2 [44] and were separated into two main steps: variable selection for predicting clinical micro-skills in our sample and the investigation of the stability of these effects with a bootstrapping algorithm.

Initial analyses included calculating correlations between the two clinical micro-skills measures (in standardized videos and in sessions) to see how closely these concepts are related and correlational analyses of all potential predictors to check for multicollinearity.

In our data, novice students were nested in seminars. To account for this hierarchical data structure, we used a multilevel approach for the prediction models. First, we calculated multilevel hierarchical linear unconditional models to estimate within- and between-seminar variability in both clinical micro-skills:

$$Clinical\;micro-skills=\gamma_{00}+[u_{00s}+r_{st}]$$ (1)

Clinical micro-skills in standardized situations (measured with an adapted version of the FIS performance task), as well as clinical micro-skills in sessions (measured with the ITIS), rated for novice students *t* and seminar *s*, were modeled as a function of the sample’s intercept (γ00), as well as a Level 2 residual (u0t—representing between-seminar variability), and a Level 1 residual (r_st_—representing within-seminar variability), see Eq 1. These models’ results provided estimates of random effects variance components, which were used to calculate intraclass correlations (ICCs) that indicate the percentage of variance in ratings explained at each level (see Boswell et al. [45] for a similar approach). Although the sample size is not large enough to allow interpretation of between-seminar variability [46], the multilevel approach still accounts for confounding effects between levels.

Previous literature on therapist characteristics suggested non-linear associations between characteristics and outcome or skills as a possible explanation of heterogeneous findings. Accordingly, bivariate scatterplots for all potential predictors were generated to examine the shape of the associations with both competence measures (S1 Fig in S1 File) [5, 47]. Based on the visual inspection of the scatterplots, non-linear associations were assumed between clinical micro-skills in standardized situations and agreeableness (quadratic), conscientiousness (cubic), empathy (quadratic), and mindfulness (cubic). Two separate regression models were calculated and compared to predict interpersonal variability in clinical micro-skills. The first model included all 14 predictors with linear associations with clinical micro-skills in standardized situations. The second model assumed quadratic associations between agreeableness and empathy and clinical micro-skills in standardized situations, and cubic associations between conscientiousness and mindfulness and clinical micro-skills in standardized situations. All other 10 predictors were assumed to be linearly associated with clinical micro-skills in standardized situations. However, comparing both models showed smaller AIC, BIC, and loglikelihood for the linear model than for the non-linear model, so the linear model was chosen for the following model predicting clinical micro-skills in standardized situations.

For clinical micro-skills in sessions, scatterplots indicated non-linear associations with agreeableness (log), conscientiousness (log), and empathy (log). The non-linear model showed smaller AIC, BIC, and logLikelihood than the first model with all linear associations so the non-linear model was chosen for the following regression.

Subsequently, to select from the potential predictors of clinical micro-skills two separate LASSO (least absolute shrinkage and selection operator) regressions were calculated. LASSO regressions are based on a machine learning algorithm and use shrinkage as a regularization method encouraging simple and more accurate regression models. Variable selection was performed with the R package glmmLASSO [48]. It includes an L 1-penalty term that enforces variable selection and shrinkage simultaneously. All potential predictors were z-standardized to facilitate interpretation. Based on a sequence of lambdas, the lambda with the smallest error (based on the BIC) was chosen.

Clinical micro-skills in standardized situations were modeled as a function of the intercept (γ00), extraversion, agreeableness, conscientiousness, neuroticism, openness, introversion, compliance, competitiveness, self-insecurity, self-confidence, resilience, well-being, empathy, a Level 2 residual (u_0s_), representing between seminar variability, and a Level 1 residual representing within seminar variability (r_ts_; see Eq 2).


$$Clinical\;micro-skills\;in\;standardized\;situations=\gamma_{00}+\gamma_{01}*Extraversion+\gamma_{02}*Agreeableness+\gamma_{03}*Conscientiousness+\gamma_{04}*Neuroticism+\gamma_{05}*Openness+\gamma_{06}*Introversion+\gamma_{07}*Compliance+\gamma_{08}*Competitiveness+\gamma_{09}*Self-insecurity+\gamma_{10}*Self-confidence+\gamma_{11}*Mindfulness+\gamma_{12}*Resilience+\gamma_{13}*Well-Being+\gamma_{14}*Empathy+u_{0t}+r_{st}$$ (2)

Clinical micro-skills in sessions were modeled as a function of the intercept (γ00), extraversion, the log_10_ of agreeableness, log_10_ of conscientiousness, neuroticism, openness, introversion, compliance, competitiveness, self-insecurity, self-confidence, mindfulness, resilience, well-being, the log_10_ of empathy, a Level 2 residual (u_0s_), representing between seminar variability, and a Level 1 residual representing within seminar variability (r_ts_; Eq 3).


$$Clinical\;micro-skills\;in\;sessions=\gamma_{00}+\gamma_{01}*Extraversion+\gamma_{02}*log(Agreeableness)+\gamma_{03}*log(Conscientiousness)+\gamma_{04}*Neuroticism+\gamma_{05}*Openness+\gamma_{06}*Introversion+\gamma_{07}*Compliance+\gamma_{08}*Competitiveness+\gamma_{09}*Self-insecurity+\gamma_{10}*Self-confidence+\gamma_{11}*Mindfulness+\gamma_{12}*Resilience+\gamma_{13}*Well-Being+\gamma_{14}*log(Empathy)+u_{0t}+r_{st}$$ (3)

Due to the inconsistency of findings in previous literature and the limited sample size, a bootstrapping algorithm implemented in the R package lmeresampler [49] was used to estimate standard errors and confidence intervals for all potential predictors. Thus, the algorithm allows evaluation of the variability and consistency of the test statistics despite the small sample size (*N* = 44). Following recommendations by Carpenter et al. [50], a residual bootstrap was implemented to create 10,000 bootstrap samples. The model was then refitted to each of the bootstrap samples and the model parameters were calculated.

## Results

### Clinical micro-skills in standardized situations

On average, novice students’ FIS total score was 2.69 (*SD* = 0.39, *range* = 1.78–3.84). The scores on the FIS domains varied between 2.04 (*SD* = 0.92, *range* = 1–4.5) on the alliance rupture-repair responsiveness domain and 3.23 (*SD* = 0.65, *range* = 2–4.75) on the verbal fluency domain. The mean levels of clinical micro-skills for all seminars are presented in S1 Table in S1 File. Average clinical micro-skills did not vary between seminars (*F*(1,42) = 2.27, *p* = .14).

The correlation between clinical micro-skills in standardized situations and clinical micro-skills in sessions was positive but non-significant (*r* = .16, *p* = .30).

### Clinical micro-skills in sessions

Novice students showed medium to satisfactory clinical micro-skills in the treatment of the simulated patients (*M* = 2.84, *SD* = 0.94, *range* = 1–4). The mean levels of clinical micro-skills for all seminars are presented in S1 Table in S1 File. Average ratings did not change over the course of the five consecutive treatment sessions (session means range = 2.67–3.00; *F*(1,42) = 0.19, *p* = .664), but varied significantly between seminars (*F*(1, 42) = 7.55, *p* < .05).

### Within and between-seminar variability

Table 2 shows the variance components for clinical micro-skills with most of the variance within seminars (i.e., between novice students). For clinical micro-skills in standardized situations, 0.1% of the total variance could be attributed to the between-seminar level. For clinical micro-skills in sessions, 17.5% of the total variance was attributable to the between-seminar level.

**Table 2:** Variability of therapeutic skills within and between seminars.

	Clinical Micro-Skills in Standardized Situations			Clinical Micro-Skills in Session		
	Variance component	SD	VPC	Variance component	SD	VPC
Between seminars	0.001	0.014	0.1%	0.156	0.394	17.5%
Within seminars	0.148	0.385	99.9%	0.734	0.857	85.5%

VPC = variance partition coefficient, the proportion of the total variance that is attributable to between seminars or within seminar variation.

### Prediction of clinical micro-skills

The results of the correlational analyses of the predictors are depicted in S2 Table in S1 File. The variance inflation factor was below five for all predictors, suggesting no multicollinearity issues in the model estimation [51]. Bivariate scatterplots were calculated for each predictor and both skills measures separately (S1 Fig in S1 File).

For clinical micro-skills in standardized sessions, the final model included extraversion (*b* = 0.10) and resilience (*b* = 0.09) as predictors. All other predictors’ weights were shrunk to zero. The final model accounted for 15% of the total variance in clinical micro-skills. For clinical micro-skills in sessions, of the 14 predictors entered into the LASSO model, none were significant. The final model included 13 predictors, namely extraversion (*b* = -0.10), log(agreeableness) (*b* = 0.03), log(conscientiousness) (*b* = 0.34), neuroticism (*b* = 0.06), openness (*b* = -0.21), introversion (*b* = -0.30), compliance (*b* = -0.01), self-insecurity (*b* = -0.01), self-confidence (*b* = 0.13), mindfulness (*b* = -0.39), resilience (*b* = 0.04), well-being (*b* = 0.17), and log(empathy) (*b* = -0.01). The coefficient of competitiveness was shrunk to zero. The final model accounted for 34% of the total variance in clinical micro-skills. Results of the LASSO models are presented in Table 3.

**Table 3:** LASSO regression models for therapeutic competence.

		Estimate	SE	*p*
Clinical Micro-Skills in Standardized Situations	Intercept	2.69	0.16	< .001
Marginal R^2^ = .16	Extraversion	0.10	0.16	.53
Conditional R^2^ = .20	Resilience	0.09	0.16	.59
Clinical-Micro-Skills in Sessions	Intercept	2.83	0.17	< .001
	Extraversion	−0.1	0.21	.65
	log(Agreeableness)	0.03	0.26	.90
	log(Conscientiousness)	0.34	0.18	.06
	Neuroticism	0.06	0.29	.83
	Openness	−0.21	0.26	.41
	Introversion	−0.30	0.22	.17
Marginal R^2^ = .34 Conditional R^2^ = .43	Compliance	−0.01	0.22	.95
	Self-insecurity	−0.01	0.23	.98
	Self-confidence	−0.13	0.21	.53
	Mindfulness	−0.39	0.33	.23
	Resilience	0.04	0.24	.85
	Well-being	0.17	0.18	.36
	log(Empathy)	−0.01	0.27	.96

Predictors with coefficients shrunk to zero are not depicted. R^2^ was calculated with the MuMIn package [52].

**Fig 2:**
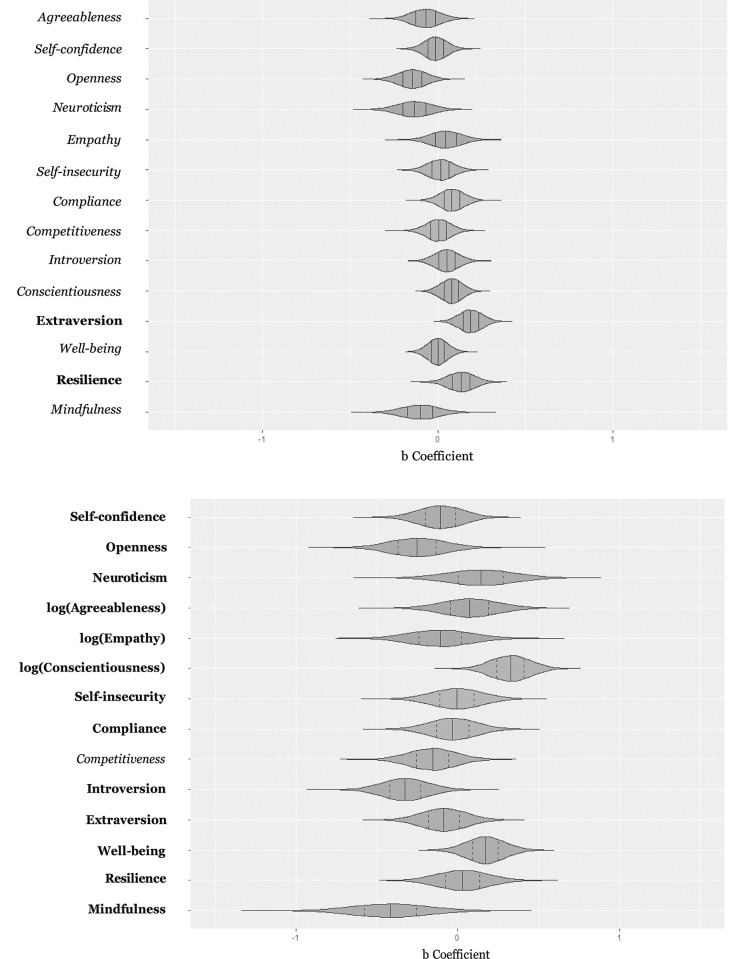
Distribution of regression coefficients across all 10,000 iterations. Vertical lines in the violin plots indicate the median and 25% quartiles. Predictors excluded by the LASSO regression are printed in italics. **a) Skills in Standardized Situations, b) Skills in Sessions**.

Results of the 10,000 bootstrapping iterations for the regression models are shown in S3 Table in S1 File. Fig 2 displays the distribution of regression coefficients across all iterations. The length of the violin plots for each predictor indicates the range of regression coefficients across all 10,000 bootstrapping iterations. The body of the violin plot represents the density of these coefficients. Wider sections represent a higher probability that the regression coefficients will take on the given value, whereas thinner sections represent a lower probability. Thus, if the shape of the distribution is extremely thin on each end and wide in the middle, this indicates a high concentration around the median, representing less variability across bootstrapping iterations. When comparing the plots for both skill measures, it is noticeable that for clinical micro-skills in standardized situations, the shapes of the distributions are more concentrated around the median than for clinical micro-skills in sessions. This indicates that predictors of clinical micro-skills in sessions show more variation across bootstrapping iterations and therefore less stable results. For both skills, many regression coefficients’ distributions include values in the positive and negative range, indicating that the direction of associations varies between iterations. For example, self-insecurity as a predictor of clinical micro-skills has a median around zero, indicating that in 5,000 iterations there was a negative association with clinical micro-skills in sessions and a positive association in the other 5,000 iterations (Fig 2B).

When comparing the predictors of clinical micro-skills in standardized situations regarding whether they were chosen by the LASSO algorithm or shrunk to zero, it is noticeable that the resilience and extraversion distributions are strongly centered around the median and show only a small proportion of negative values, indicating a relatively stable positive association with clinical micro-skills in standardized situations (Fig 2A).

To summarize, the violin plots of the regression coefficients’ distributions across bootstrapping iterations can be used to evaluate the variability and stability of predictors that were chosen by the LASSO regression. For clinical micro-skills in standardized situations, the two predictors chosen by the LASSO algorithm showed distributions that were more centered, less wide, and had a clearer direction, indicating that the algorithm chose the two predictors with the most stable results across all bootstrapping iterations. For clinical micro-skills in sessions, the LASSO algorithm was not able to find a parsimonious model with significant predictors but included 13 of 14 predictors, which is also reflected in the violin plots that showed high variation in the direction of associations and range of all coefficients.

## Discussion

The present study aimed to investigate the associations between therapist characteristics and the initial clinical micro-skills of novice students working with simulated patients. A multilevel LASSO regression and a bootstrapping algorithm with 10,000 iterations were used to select predictors and examine their stability. Clinical micro-skills were assessed based on video ratings and included clinical micro-skills in reaction to standardized situations with simulated patients and clinical micro-skills during a session with a simulated patient. Further, non-linear associations between therapist characteristics and in-session-skills were examined if scatterplots indicated non-linearity.

The first research question guiding this study was: What characteristics of novice students are associated with clinical micro-skills in standardized situations? The variable selection algorithm found a parsimonious prediction model with extraversion (*b* = 0.10) and resilience (*b* = 0.09) predicting clinical micro-skills in standardized situations. However, both regression coefficients were not significantly associated with the clinical micro-skills in standardized situations. Looking at the results of our bootstrapping method (a resampling method), it became clear that compared to the other characteristics we considered (which were eliminated by the LASSO method), extraversion and resilience showed less variability across the 10,000 iterations. This could be an indication that in a larger sample, extraversion and resilience could be significantly and positively related to clinical micro-skills whereas this is very unlikely for the other predictors showing higher variability in the bootstrapping procedure. It is important to emphasize that having a larger sample size does not solve everything. This point is underscored by a recently published study with a sample of n = 177 novice and advanced students. In that study, the authors did find a connection between skills in the FIS task and certain factors like greater experience, male gender, and lower levels of alexithymia. However, they did not find associations with personality-related characteristics such as emotion regulation, attachment style, or self-concept variables [53]. Nevertheless, despite their lack of significance in the final model, previous literature has provided first evidence that extraversion and resilience could be promising characteristics to investigate in future studies. For example, in a study by Pereira and colleagues (n = 37), effective therapists showed a combination of higher resilience and mindfulness than their less effective colleagues [54]. The authors argue that in the general population, extraversion is associated with optimism, confidence, positive affect, and well-being so it might positively influence professional growth. Furthermore, extraversion has been found to be associated with *Healing Involvement* in therapists in training which comprises basic relational skills, the experience of agency, affirmative relational style, and constructive coping [55]. Additionally, in our study, novice students were asked to react to short video clips of simulated patients, which can be perceived as a stressful task, as students are not used to these kinds of performance-based tasks in their studies. It is possible that this task is less intimidating for students who are more extroverted and hence feel less stressed, leading to better performance. A higher level of resilience could also help students cope with the stress induced by the task and enable them to show more skills. The novice students in our sample were at the beginning of their professional development in the novice student phase according to Rønnestad and Skovholt’s [18] model. This phase is characterized by initial patient contact triggering intense emotions, leading to more preoccupation with themselves and a predominantly internal focus. The intensity of these reactions may be so profound that even individuals with a generally heightened level of mindfulness may struggle to effectively manage them. Consequently, it is possible that novice students with a higher baseline of mindfulness, do not necessarily outperform their peers with lower levels of mindfulness in handling these reactions. Based on Pereira and colleagues’ [54] findings, we would expect to find an effect of mindfulness on clinical micro-skills in standardized situations in therapists’ later development. When therapists have more experience and can shift their focus to the interpersonal aspects of a therapy situation, having already practiced mindfulness in their everyday lives should be an advantage. However, due to the non-significant regression coefficients in our sample, these associations remain hypothetical and have to be tested in separate studies.

The second research question guiding this study sought to understand the relationship between characteristics of novice students and their clinical micro-skills during simulated sessions. The variable selection algorithm did not yield significant predictors for these skills in sessions and excluded only one of the initial 14 predictors (i.e., competitiveness). Among the remaining predictors, only the logarithm (log_10_) of conscientiousness showed marginal significance (*b* = 0.34, *p* = 0.06). The results obtained through bootstrapping, revealed considerable variability across the iterations in terms of the direction of associations and the range of regression coefficients. Notably, when comparing the distributions of regression coefficients between models for clinical micro-skills in standardized situations and those in simulated sessions, a distinct pattern emerged. In the case of in-session skills, therapists’ characteristics exhibited greater variability when predicting these skills. This suggests that there were no strong individual predictors that could consistently explain the variance in clinical micro-skills during therapy sessions at the therapist level. However, the distribution of coefficients was narrower and more centered around the median for the log10 of conscientiousness, aligning with its marginally significant regression coefficient. This is in line with a previous study on therapists in training which found that conscientiousness was negatively correlated with *Stressful Involvement*. This correlation was interpreted as indicating that conscientiousness serves as a protective factor against avoidant coping strategies [55]. Our findings hint that this protective effect may not increase linearly but might plateau at a certain level of conscientiousness. One plausible explanation is that excessively high levels of conscientiousness could lead to counterproductive behaviors, such as working excessively long hours or exhibiting perfectionist tendencies. This underscores the importance of considering individual variations in the associations between therapist characteristics and competence or outcomes. However, it is essential to note that the regression coefficient for this observation was not statistically significant. Consequently, these theoretical considerations warrant further exploration within a larger sample size.

The variability in regression coefficients detected by the bootstrapping algorithm is in line with the heterogeneity of previous findings when trying to identify therapist characteristics as predictors of therapeutic skills or treatment outcome (for a summary, see Wampold & Owen [11]).

Two methodological explanations of this heterogeneity in predictors of clinical micro-skills in sessions could be: a) that novice students’ clinical micro-skills in standardized situations were aggregated over more than one simulated patient video, which could result in stronger associations with personal characteristics than the isolated work with only one patient (which was the case for clinical micro-skills in sessions); and b) since the seminar was elective, it is possible that the sample of psychology students was selective (e.g., only students that were less self-insecure and more outgoing), resulting in limited variance in our predictors. Additionally, students’ competence ratings were at a medium level, which was appropriate for their stage of professional development, but did not depict the whole range of the scale (*M* = 2.84, *SD* = 0.94, ranging between 1 and 4, whereas the scale ranges up to 6). Thus, it is possible that therapist characteristics can explain differences between highly and moderately competent therapists, but not between novice students who were rated at a similar level.

A more clinical interpretation could be that one difference between the two aspects of clinical micro-skills (i.e., in standardized situations vs. in session) was the type of interaction between novice students and simulated patients. The spontaneous reactions elicited by a video featuring a simulated patient may be influenced differently by personal characteristics compared to the planning and execution of a full therapy session. In the latter case, various factors such as experience, current learning state, and feedback from supervisors could exert a more pronounced impact. This distinction is highlighted by the non-significant correlation between the two measures of clinical micro-skills (*r* = .16, *p* = .30), suggesting that each measure assesses different facets of skills. Furthermore, it is essential to consider that while the standardized video clips presented particularly challenging scenarios, the instructions given to simulated patients were designed to keep the case complexity low. Differences in the perceived difficulty of these two skill assessments might have contributed to the varying results, with performance in more difficult scenarios being influenced more by personal characteristics than low-difficult scenarios. However, it is noteworthy that the variance in clinical micro-skills during therapy sessions was higher than that observed in the FIS task (see S1 Table in S1 File), which partially contradicts this hypothesis.

These contrasting outcomes raise a critical question about the equivalency of both measures in assessing therapy-relevant skills in novice students. Existing research suggests that therapists with higher FIS scores tend to be more effective in short-term therapies [27]. Additionally, previous studies have established the authenticity of simulated patients [31], their positive impact on students’ self-efficacy [30] and self-rated competence [33]. Nevertheless, to our knowledge, no prior research has explored the association between simulated patient interactions and subsequent clinical skills or therapy outcomes. The efficacy of simulated patients as a training method warrants investigation in future prospective studies [56].

Another difference between the two measures is that during the simulated therapy sessions, patients responded to therapists’ reactions, thus co-determining how the sessions developed. In line with this idea, clinical micro-skills in sessions varied significantly between seminars, whereas clinical micro-skills in standardized situations did not. Accordingly, 17.5% of the variance in clinical micro-skills in sessions was at the between-seminar level, all of which treated different simulated patients and were supervised by different supervisors, whereas only 0.1% of the variance in clinical micro-skills in standardized situations was at the between-seminar level. Previous studies have examined patient characteristics in relation to the therapist effect (for a summary see Constantino, Boswell, & Coyne [57]) and focused on the match between patient and therapist [58], highlighting the importance of combining intra- and inter-personal aspects to understand what happens in therapy. Furthermore, supervisors could have had a high impact on the development of their novice students’ clinical micro-skills in sessions in our study, partially accounting for the variance between seminars. Accordingly, a recent meta-analysis found that supervision accounted for 4% of variance in client outcomes [59]. This underlines the importance of understanding more about supervisors’ competence and the processes involved in supervision to better understand therapists’ skill acquisition.

It is likewise conceivable that some therapist characteristics cannot explain differences in clinical micro-skills in sessions at this early stage of professional development but affect learning patterns and hence gain influence later on. This aspect has already been discussed regarding professional self-doubt, which has been found to be associated with decreased interpersonal problems in experienced therapists’ patients [9], but more interpersonal problems in psychotherapist trainees’ patients [10]. Thus, it is possible that at different stages of a psychotherapist’s career, different characteristics are beneficial to master the corresponding challenges [18].

### Strengths, limitations, and future research

We believe that the present study has several strengths and contributes to the literature on therapist competence and therapist training. Firstly, the results of this study are derived from video-based competence ratings with two different measures focusing on different aspects of clinical micro-skills. Previous findings have indicated that results of individual aspects of competence are not generalizable to other aspects of competence, which highlights the importance of differentiated assessment. Applying two different measures to the same sample could thus help to better understand the heterogeneous findings in this line of research. To our knowledge, the present study is the first to apply two different video-based ratings of therapeutic skills to find associations with therapist characteristics.

Concerning the statistical approach, applying multilevel models can control for the nested structure of the data. By examining the descriptive shape of characteristics-competence associations, non-linear relations could be detected that might have been overlooked if linear relations were assumed for all predictors. Including non-linear relations improved the prediction model for clinical micro-skills in sessions. This is in line with a study by Delgadillo and colleagues [5] that found a non-linear relation between above-average expression of agreeableness and outcome. This could indicate that it makes a difference in therapist skills when some characteristics are below average versus average, but there are no further benefits to an above-average level of the characteristic (i.e., logarithmic). Further research should focus on detecting these non-linear relations and thereby contributing to the overall pattern of therapist characteristics- competence- outcome associations.

Finally, and most importantly, due to the applied machine learning approaches (i.e., LASSO regression and bootstrapping), a variable selection procedure was conducted and the stability of the results could be examined. This approach takes the heterogeneity of previous findings into account and helps draw appropriate interpretations from the results.

Although using simulated patients creates a safe setting for students to practice without the risk of harm, a drawback could be that it is unclear whether our findings can be generalized to real therapy situations. Previous studies have shown that this setting is realistic and comparable to “real world” therapies [30, 31]. Yet, the results apply only to student therapists at the novice student stage and cannot be generalized to more experienced therapists conducting “real” therapy. Additionally, the novice students in our sample were taught within a CBT framework, which could question the generalizability of findings to other therapy orientations. However, we would not expect the findings to be specific to CBT approaches, as the micro-skills investigated in this study–opposed to specific techniques—are basic clinical skills that are shown across all orientations [e.g., see 60, 61 for common factor approaches to clinical training]. As process research has shown, therapists contribute to several global change mechanisms (e.g., establishing working alliance, fostering motivation [11], which could mediate better therapy outcomes. To deepen our understanding, future studies should expand their focus and investigate these change mechanisms, their interrelations with therapist characteristics and skills, and their association with outcome within and across therapy orientations.

Another limitation is the small sample size (*N* = 44). Applying a bootstrapping algorithm with 10,000 iterations to our prediction models allowed us to construct confidence intervals but cannot perform bias corrections of small sample sizes. Concerning the variance components, it is important to mention the sample size is not sufficient to make valid and reliable estimations. For a more valid and reliable estimation of within and between novice students’ associations, multiple assessments are needed [62]. Further, to make valid estimations of the variance components within and between novice students or seminars/ supervisors, recommendations for finding therapist effects should be applied, because small samples usually overestimate between-therapist variability [46]. For our sample, this would indicate that each student (*N* = 44) should treat at least four simulated patients to make reliable estimations of student-therapist effects. Additionally, each supervisor (*N* = 7) would have to supervise at least 15 students to draw reliable interpretations of the estimated between-supervisor variance. Future studies should take these sample size considerations into account when planning to examine therapist characteristics. Having novice students treat more than one simulated patient would provide valuable training opportunities, as well as the possibility for research to investigate intra-individual progress and learning processes. Examining larger samples could also facilitate the differentiation of competencies by including subscales of competence measures in the analyses and thus gaining a more profound understanding of competence profiles in therapists. Therapist training could be personalized based on these profiles of individual strengths and weaknesses. One way of doing this could be to give competence feedback to therapists, which has already been shown to improve competence over the course of treatment in a randomized controlled trial with 67 therapist trainees treating 114 depressed patients [63]. Additionally, this feedback could be paired with individual training opportunities, for example, deliberate practice. Deliberate practice is defined as repeated training with feedback [64]. It has been identified as beneficial for professional development across several domains, recently also including psychotherapy training, where a significant association between the amount of time spent improving therapeutic skills and patients’ outcomes has been found [3]. Using deliberate practice workshops in contrast to traditional workshops in continuing education has been successful in improving specific skills, for example managing patients’ resistance [65]. The implementation of deliberate practice paired with routine outcome monitoring was associated with improved effectiveness within an agency [66], highlighting the promising potential of this emerging training method, both for individual therapists and institutions.

A last limiting factor is the time- and cost-intensity of video-based ratings, which is reflected in the small sample size. Thus, automatized skills ratings could play a major role in driving this line of research forward. For example, Goldberg and colleagues [67] used automatic speech recognition software to predict alliance ratings from session content, underlining the potential of such automated ratings for the investigation of important process variables or the implementation of feedback-based training in the future.

### Clinical implications

It is important to acknowledge the limitations of this study; however, the congruence of our results with existing literature highlights the necessity of considering the clinical implications that emerge from our findings. First, our results suggest that novice students’ resilience could be associated with their clinical micro-skills in standardized situations, which is in line with previous research. Thus, researchers have increasingly attempted to determine which personal or context factors can enhance resilience in therapists to increase their effectiveness. One important aspect associated with therapists’ resilience seems to be the workplace setting (e.g., independent practitioners vs. practitioners working for an agency) [68]. Further, self-efficacy beliefs and having a purpose or meaning in their daily work have been identified to be positively associated with therapists’ resilience [69], and self-care may reduce stress, leading to less burnout and greater life satisfaction [70]. Besides teaching professional knowledge and therapeutic skills, therapist training should also focus on personal development and train novice students to proactively focus on their own well-being [69]. While Skovholt and Trotter-Mathison [71] provide comprehensive information on resilience and self-care strategies for novice students in their book, to our knowledge no such program has been developed or tested for therapists. Future research on therapist training could close this gap and test whether targeted resilience training can improve clinical micro-skills.

Further, by focusing on novice students’ individual profiles of strengths, personality traits, and weaknesses, training could be tailored to individual needs. For example, in our study, higher extraversion was identified as potentially associated with better clinical micro-skills in standardized situations. If this finding replicates in a study with a larger sample, it could be useful to explore whether less extroverted novice students might benefit from specifically adapted training. Assessing therapist characteristics throughout clinical training could help to find the appropriate training modules for each therapist.

Second, supervisors (or trainers) could play an important role in the development of therapists’ skills and in recent years, the focus on supervisor training has increased [72]. Further research is needed to understand the underlying processes of supervision and empirically support supervisor training.

## Conclusions

Our study indicates that novice students who are more extraverted and resilient tend to perform better in clinical micro-skills when reacting to short video clips of simulated patients. We could not identify indicators that consistently predicted how well therapists performed in clinical micro-skills when interacting with simulated patients. When we looked at the regression coefficients across 10,000 different scenarios (bootstrapping iterations), we noticed that the ability to predict clinical micro-skills varied. This is in line with the heterogeneous results reported by previous studies on therapeutic characteristics and skills.

Our study underscores the need to distinguish between different aspects of competence (i.e., reactions to videos vs. role-plays vs. “real” therapy) and different stages of professional development in the therapist training. From a methodological perspective, our study emphasizes the significance of incorporating multiple competence ratings and multilevel approaches to derive accurate estimations of variance components when therapists are nested within supervisors. Taking into account non-linear relationships when studying therapist characteristics may help reduce variability in findings.

To enhance therapeutic competence and outcomes in psychotherapy, we suggest incorporating resilience workshops into therapist training, as well as feedback on therapeutic skills profiles that could be implemented in comprehensive feedback systems [73]. Therapists’ strengths and weaknesses could be identified to personalize psychotherapy training to therapists’ individual characteristics and needs, leading to evidence-based education. Additionally, it is important to consider the influence of supervisors when examining novice student samples. Future research should explore how therapists’ learning processes and specific training methods, like deliberate practice, can enhance competence and effectiveness. To maintain the reliability of video-based ratings for research, it is important to further explore automated competence assessment using machine learning algorithms so that feedback and training methods can be efficient and applicable for everyday use.

## Supporting information

S1 File (DOCX)
