# Supplementary material for: Where it all begins: Predicting initial therapeutic skills before clinical training in cognitive behavior therapy
Source: PLoS One. 2024 Feb 22;19(2):e0294183. doi: 10.1371/journal.pone.0294183 (PMC10883575; doi:10.1371/journal.pone.0294183)
Supplement: S1 File — (DOCX) [file pone.0294183.s001.docx]

**Supporting Information**

**Table S1. Therapeutic Skills across Seminars.**

|  | Seminar 1 *M* (SD) | Seminar 2 *M* (SD) | Seminar 3 *M* (*SD*) | Seminar 4 *M* (*SD*) | Seminar 5 *M* (*SD*) | Seminar 6 *M* (*SD*) | Seminar 7 *M* (*SD*) |
| --- | --- | --- | --- | --- | --- | --- | --- |
| Skills in Standardized Situations | 2.65 (0.30) | 2.58 (0.48) | 2.74 (0.42) | 2.53 (0.24) | 2.84 (0.16) | 2.63 (0.28) | 2.91  (0.50) |
| Skills in Sessions | 2.75 (1.28) | 2.44 (0.73) | 2.14 (0.69) | 3.00 (0.82) | 2.50 (0.58) | 3.00 (1.00) | 3.75  (0.71) |

Differences between seminars were significant for in-session-skills (*F*(1, 42) = 7.55, *p* = .008), but not for skills in standardized situations (*F*(1,42) = 2.27, *p* = .14).

**Table S2. Correlation Matrix for Possible Predictors of Therapeutic Skills.**

| Correlations | Empathy | Well-being | Resilience | Extraversion | Agreeableness | Conscientiousness | Neuroticism | Openness | Introversion | Compliance | Competiveness | Self-insecurity | Self-confidence | Mindfulness |
| --- | --- | --- | --- | --- | --- | --- | --- | --- | --- | --- | --- | --- | --- | --- |
| Mindfulness | -.035 | -.247 | .505** | .278 | .096 | .369* | -.593** | .205 | -.444** | -.632** | -.431** | -.617** | .213 | 1 |
| Self-confidence | .103 | -.367* | .328* | .117 | .195 | -.090 | -.242 | .413** | -.072 | -.097 | -.176 | .077 | 1 |  |
| Self-insecurity | .250 | .090 | -.283 | -.289 | .085 | -.219 | .385** | -.096 | .123 | .464** | 237 | 1 |  |  |
| Competiveness | -.336* | .290 | -.528** | -.146 | -.240 | -.252 | .568** | -.421** | .206 | .193 | 1 |  |  |  |
| Compliance | .145 | .066 | -.329* | -.316* | -.024 | -.289 | .295 | -.040 | .356* | 1 |  |  |  |  |
| Introversion | -.057 | .215 | -.327* | -.485** | -.110 | -.261 | .274 | -.348* | 1 |  |  |  |  |  |
| Openness | .067 | -.231 | .522** | .395** | .027 | .020 | -.591** | 1 |  |  |  |  |  |  |
| Neuroticism | .054 | .265 | -.637** | .323** | -.137 | -.153 | 1 |  |  |  |  |  |  |  |
| Consientiousness | .184 | -.288 | .342* | .024 | .346* | 1 |  |  |  |  |  |  |  |  |
| Agreeableness | .682** | -.269 | .227 | -.134 | 1 |  |  |  |  |  |  |  |  |  |
| Extraversion | .275 | -.213 | .312* | 1 |  |  |  |  |  |  |  |  |  |  |
| Resilience | .239 | -.331* | 1 |  |  |  |  |  |  |  |  |  |  |  |
| Well-being | -.170 | 1 |  |  |  |  |  |  |  |  |  |  |  |  |
| Empathy | 1 |  |  |  |  |  |  |  |  |  |  |  |  |  |

*p < .05, ** p < .01

**Table S3. Bootstrapped Regression Models for Therapeutic Skills.**

|  |  | Observed | Rep mean | SE | Bias | df | *p* |
| --- | --- | --- | --- | --- | --- | --- | --- |
| Skills in | **Intercept** | 2.69 | 2.69 | 0.06 | 1.62 | 3.98 | 0.00 |
| Standardized | **Extraversion** | 0.19 | 0.19 | 0.08 | 2.54 | 27.63 | 0.03 |
| Situations | Agreeableness | −0.07 | −0.07 | 0.10 | 4.75 | 28.35 | 0.49 |
|  | Conscientiousness | 0.08 | 0.08 | 0.07 | 2.91 | 28.79 | 0.31 |
|  | Neuroticism | −0.13 | −0.13 | 0.12 | 2.60 | 28.71 | 0.29 |
|  | Openness | −0.15 | −0.15 | 0.10 | 1.43 | 28.24 | 0.16 |
|  | Introversion | 0.05 | 0.05 | 0.08 | 5.52 | 26.97 | 0.58 |
|  | Compliance | 0.08 | 0.08 | 0.08 | 3.26 | 28.98 | 0.35 |
|  | Competitiveness | 0.00 | 0.00 | 0.08 | 9.66 | 27.66 | 0.97 |
|  | Self-insecurity | 0.02 | 0.02 | 0.09 | 8.62 | 28.92 | 0.87 |
|  | Self-confidence | −0.01 | −0.01 | 0.08 | 8.70 | 28.98 | 0.87 |
|  | Mindfulness | −0.10 | −0.10 | 0.12 | 4.19 | 24.25 | 0.45 |
|  | **Resilience** | 0.13 | 0.13 | 0.09 | 1.62 | 26.70 | 0.18 |
|  | Well-being | −0.00 | −0.00 | 0.07 | 9.91 | 27.72 | 0.99 |
|  | Empathy | 0.05 | 0.05 | 0.11 | 6.70 | 25.94 | 0.68 |
| Skills in Sessions | **Intercept** | 2.81 | 2.81 | 0.17 | 2.65 | 4.63 | 0.00 |
|  | **Extraversion** | −0.09 | −0.09 | 0.17 | 6.19 | 26.90 | 0.63 |
|  | **Log(Agreeableness)** | 0.07 | 0.07 | 0.21 | 7.36 | 27.32 | 0.75 |
|  | **Log(Conscientiousness)** | 0.33 | 0.33 | 0.15 | 3.94 | 27.48 | 0.05 |
|  | **Neuroticism** | 0.14 | 0.14 | 0.25 | 5.73 | 27.98 | 0.59 |
|  | **Openness** | −0.25 | −0.25 | 0.21 | 2.42 | 27.06 | 0.27 |
|  | **Introversion** | −0.32 | −0.32 | 0.18 | 8.20 | 26.00 | 0.09 |
|  | **Compliance** | −0.03 | −0.03 | 0.18 | 8.58 | 28.40 | 0.87 |
|  | Competitiveness | −0.16 | −0.16 | 0.18 | 3.92 | 26.71 | 0.42 |
|  | **Self-insecurity** | −0.00 | −0.01 | 0.19 | 9.80 | 28.38 | 0.98 |
|  | **Self-confidence** | −0.11 | −0.11 | 0.17 | 5.36 | 28.59 | 0.56 |
|  | **Mindfulness** | −0.41 | −0.41 | 0.28 | 1.44 | 28.21 | 0.17 |
|  | **Resilience** | 0.03 | 0.03 | 0.20 | 8.71 | 25.62 | 0.88 |
|  | **Well-being** | 0.17 | 0.17 | 0.15 | 2.53 | 26.67 | 0.26 |
|  | **Log(Empathy)** | −0.11 | −0.10 | 0.24 | 6.67 | 25.08 | 0.68 |

Observed = Coefficient in standard regression; Rep mean = Regression coefficients are based on 10,000 iterations of residual bootstrap. Regression coefficients that were included in the final LASSO models are printed in bold.

**Figure S1**


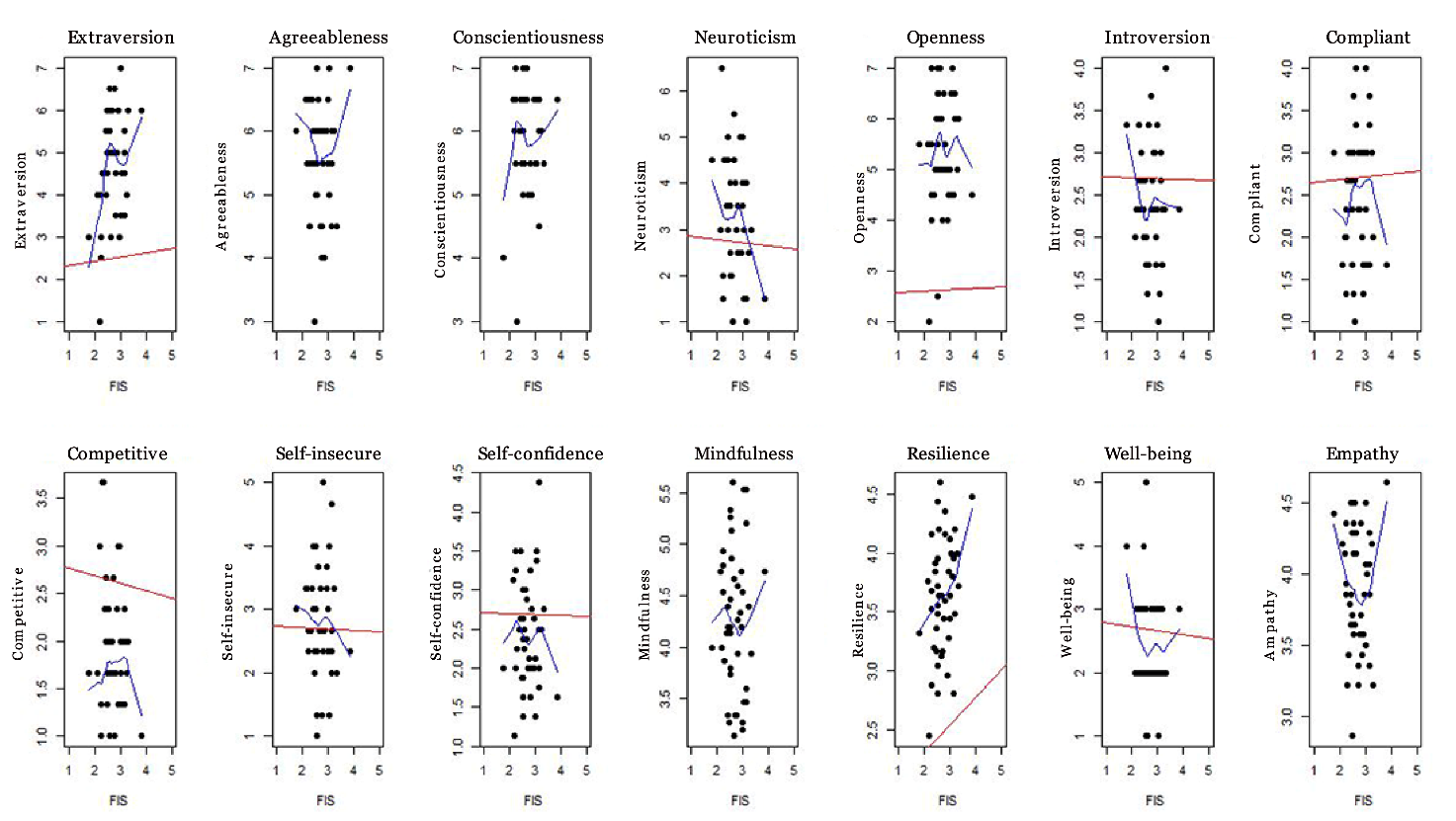


1. *Bivariate Scatterplots for Predictors and Skills in Standardized Situations*


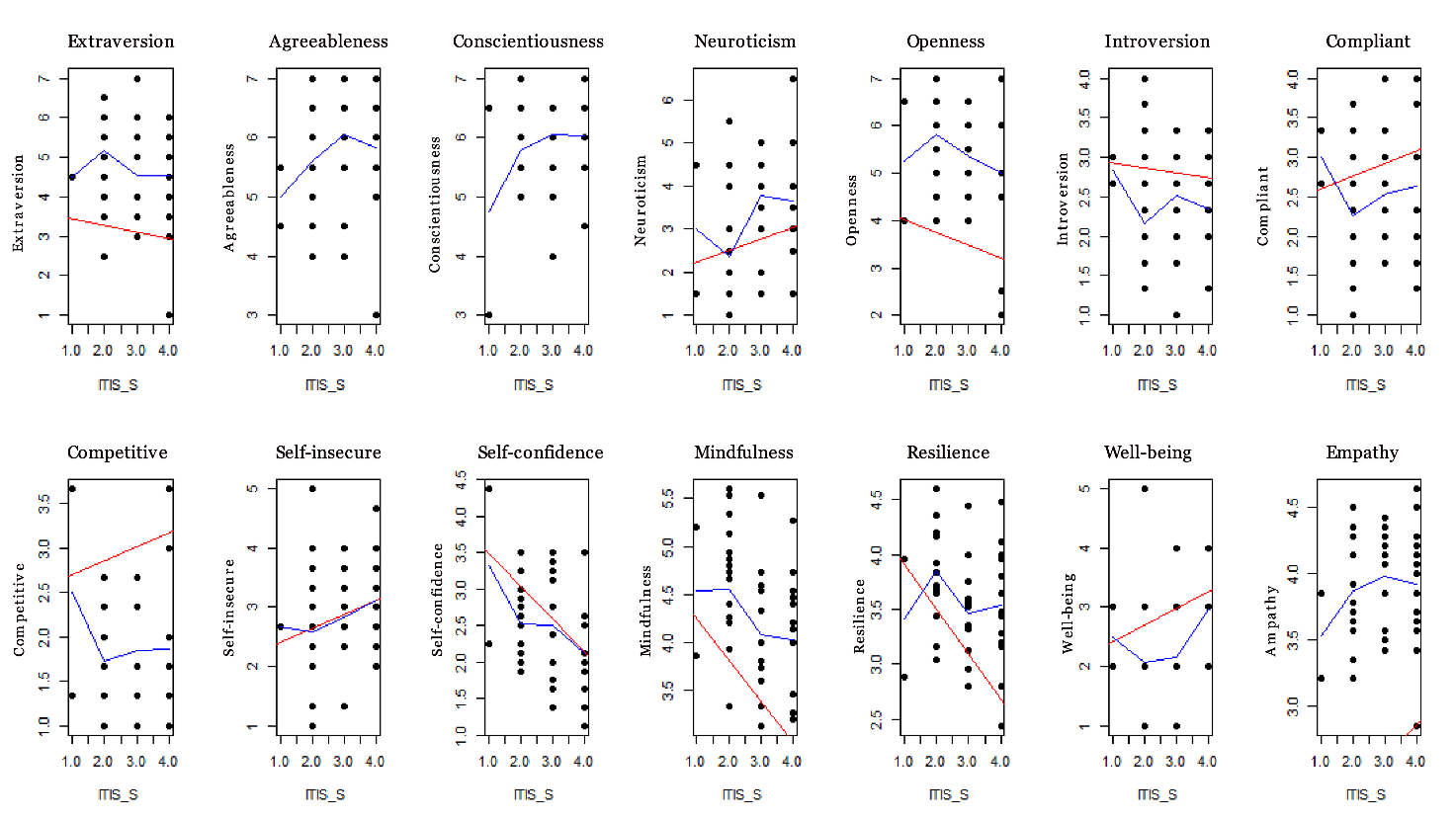


1. *Bivariate Scatterplots for Predictors and Skills in Sessions*

ITIS_S = Skills in Sessions; FIS = Skills in Standardized Situations.
